# Material Characteristics of Compressed Dry Masonry Made of Medium-Size Elements with Perlite Aggregate

**DOI:** 10.3390/ma17225406

**Published:** 2024-11-05

**Authors:** Adam Piekarczyk, Łukasz Drobiec

**Affiliations:** Department of Building Structures, Faculty of Civil Engineering, Silesian University of Technology, 44-100 Gliwice, Poland

**Keywords:** dry masonry, expanded perlite aggregate, design assisted by testing, material models for analysis, dry masonry capacity reduction factor

## Abstract

Dry masonry is a type of construction that is nowadays used to a limited extent in the construction sector, including the housing sector. A lack of codified computational methods enabling engineers to design consciously is one of the factors limiting the development of dry walls. This article presents results from testing an innovative solution for dry masonry made of medium-size elements with expanded perlite aggregate. Material with this type of aggregate has a low bulk density (390 ± 10% kg/m^3^), which allows the production of large blocks and significantly reduces the value of the thermal conductivity coefficient λ = 0.084 ± 0.003 W/m·K. The results obtained were used to determine material parameters for designing a structure mainly exposed to vertical load. The important practical significance of the presented research results from the lack of provisions, specifications or standards allowing for the design, calculation and construction of dry masonry; it is not possible to analyse the behaviour of this type of structure and to design it consciously and safely. The presented research is therefore an important source of information on mechanical parameters essential for the design of structures and provides tools for this. As a result of the tests of nine panels, the mean compressive strength was determined (1.085 N/mm^2^), and then the procedure of “design assisted by testing” implemented into Eurocode was used to determine characteristic (0.873 N/mm^2^) and design compressive strength (0.565 N/mm^2^). Using the relationships σ-ε, an attempt was made to identify material models for the linear and non-linear analysis of the structure and for designing cross-sections. The material models were made considering increased non-linear deformations of a structure under low stresses (the compression toe) which are true and typical for dry masonry. A specific deformability of dry masonry, slightly different to that in masonry structures joined together with mortar, also affects the reduction factor for load-bearing capacity due to second-order effects. Reduction factors determined from true non-linear deformations were lower than the values specified by EC6 for masonry structures.

## 1. Introduction

Dry masonry (dry stone, dry stack, drystane) is a type of structure where masonry units are laid intentionally without using mortar or any other binding material.

Originally, selected stones or bigger fragments of rocks, and later sun-dried bricks, were used as masonry units for such structures. Forces were transmitted from one masonry unit to another through friction or specially shaped surfaces of masonry units forming joints. In the oldest structures, the interlocking mechanism was applied by placing smaller stones or earth between larger blocks of rock [1].

Houses near Lake Huller, Israel (9000–8000 B.C.) are probably the oldest buildings made from dry masonry [2,3]. The walls of Jericho (8000 B.C.), built from limestone with gaps between blocks filled with earth [4], are the oldest known masonry structure of defensive type. Other well-known structures erected in the technology of dry masonry are, among others, Segovia (2nd half of 1st century A.D.) and Pont du Gard (50 A.D.) aqueducts, which still exist today [5].

Advantages and disadvantages of contemporary systems of dry masonry are usually affected by a lack of mortar joining together these elements. Potential advantages of dry masonry structures over masonry in which the masonry elements are bonded with mortar are the following:

- faster and consequently cheaper erection of such structures;
- the structure can be subjected to loading directly after its completion;
- water consumption is reduced;
- dry masonry can be built regardless of the ambient temperature;
- the effects related to wall swelling or shrinkage can be minimized;
- dry masonry can be built by less-qualified workers;
- in some regions, structural elements can be produced in situ, which enables the use of local raw materials, the reduction of transport costs, the reduction of CO_2_ emissions, and activation of local workforce.

Possible drawbacks of dry masonry that traditional masonry does not have are the following:

- a lack of mortar used as a link, which enables the relative displacements of masonry units, which can cause greater deformations. A lack of adhesion in contact planes of masonry units can have a negative impact on possible transfer of shear and tensile stresses by the structure;
- a lack of mortar used as a cushion, which increases stresses in places of their concentration and increases a number of areas of concentrated stress due to uneven contact surfaces of masonry units, which is connected with greater deformations of the masonry;
- a lack of mortar used as a barrier, so the structure tightness is smaller;
- the necessity of pre-forming masonry units with high precision with relatively low dimensional tolerance;
- elements of the system of joints between masonry units are exposed to damage during assembly and transport.

Modern dry masonry can be divided into reinforced and unreinforced structures. The unreinforced structures are usually made of solid masonry units without any openings or chases, which allow the potential use of rebars, as in the case of the construction system described in this paper (however, superficial reinforcement can be also applied). Masonry units, from which reinforced structures are built, usually have vertical openings, and sometimes horizontal chases, which can be used for embedded reinforcement. In the above case, filling mortar is required to provide the interaction between masonry units and reinforcement. Vertical openings can be also filled with mortar and reinforcement is not required. This technology of erecting reinforced masonry eliminates nearly all mentioned advantages of dry masonry, and at the same time eliminates many disadvantages.

The Canadian Haener Block system [6,7] is one of the systems used to build reinforced masonry from concrete masonry units. Other similar systems are the Mecano System [8], in which calcium-silicate masonry units are used, the Lok Bild System [9], the H-Block and W-Block Systems [10], while vertical joints in the W-Block system have a stack bond pattern. Masonry units made from aggregate concrete are used in H-Block and W-Block systems. In the Sparlock system [11], concrete elements are laid in simple dry stacked technology, and mortar is used to fill vertical openings or surface bonding is applied. Surface bonding is also used in the Sparfill system [12], with masonry units made from lightweight concrete. Concrete elements are used in the Putra Block system [13].

Some structures are post-tensioned by means of vertical steel tendons [14]. The Interlocking Hollow Block System [15] does not require reinforcement. However, one of its development versions had vertical openings for vertical reinforcement [16]. Solid Inter-Locking Block (Silblock) [17,18] is another system which does not need reinforcement. Bed joints in the Silblock system are not continuous as masonry units made from foam concrete have different heights (there is a bond in the horizontal direction).

The Interlocking Stabilised Soil Blocks (ISSB) system belongs to the systems of dry masonry, which is mainly used in developing countries. In this technology, masonry units are made from local raw materials, such as clay soil, murrum soil (soil of humid tropical or equatorial zones), and sand. This mixture is stabilized with cement or lime. Elements are formed in hand or hydraulic presses [19,20,21].

Wall panels, whose individual layers are not joined with binder, are made from other materials, e.g., wood materials [22] and plastic [23].

Published results from tests on mechanical parameters of dry masonry refer to compressive strength under axial load exerted on single elements, dry stacks and panels assembled from these elements, and relationships between these strengths [24,25,26,27]. Compressed panels were analysed with FEM [28,29,30], including the method of homogenization [31]. The paper [32] described tests on dry stacks under axial and eccentric compression. A drop in load-bearing capacity within a range of 11–36% was caused by a relative eccentricity of loading equal to t/6. The paper [18] also presented results from testing dry stacks under eccentric compression. For load on the top surface of dry stacks under eccentricity of t/6, the achieved strength was equal to 0.42–0.55 of the strength of dry stacks under axial compression. Under eccentric load on both sides of dry stacks—eccentricity of t/3 at the top surface and t/6 at the bottom surface—the strength of dry stacks was approx. 0.25 of the strength of dry stacks under axial compression. Some papers mentioned greater deformations of structure under relatively low compressive stress resulting from irregularity and mismatching of element surfaces, through which compressive stresses were transmitted to other layers [25,33,34,35,36] and from differences in block heights [37,38]. Methods of minimizing effects of mismatching of blocks were analysed in [25,39]. Stress distribution between blocks of the masonry under concentrated force [40] was also tested.

Tests were conducted on the behaviour of walls under in-plane [5,33] loading, including post-tensioned [41] and confined [42] shear walls. Structures under out-of-plane loading [11,17,43], including reinforced [44] and post-tensioned [45] walls, were also analysed. These cases were also subjected to numerical analyses [46,47]. Tests were also performed on walls subjected to soil settlement [48].

The publications cited above determined some parameters of various types of dry walls and drew attention to increased deformations of dry masonry resulting from block mismatches. However, according to the authors, the real engineering challenge is to use scientific research in the service of practical applications. The results of experimental studies should serve as a contribution to the process of codifying the principles of dry masonry design and provide tools for this. Usually in the design regulations, in addition to strength and deformation parameters, mathematical models of materials are also provided, which are used for more or less advanced analysis of the behaviour of the structure under load. The standard regulations provide information on methods of checking the load-bearing capacity and on the construction conditions. In the case of dry masonry, it is difficult to find documents containing the above information. Not all methods of checking the load-bearing capacity can be transferred to dry masonry, for example from standards on the design of masonry structures. This applies, among others, to considering larger deformations of dry masonry walls affecting second-order effects and coefficients reducing the load-bearing capacity of an eccentrically compressed structure. For these reasons, the main objective of tests and analyses mentioned in this paper was to provide information to design wall structures from medium-size innovative masonry units made of concrete with expanded perlite aggregate erected without mortar.

It should be clearly emphasized that Eurocode 6 refers to design of masonry structures, and according to the term specified in the standard EN 1996-1-1 [49] (p. 3.1.1.1) the masonry structure is an “assemblage of masonry units joined together with mortar”. On the other hand, according to point 1.1 (6) of the standard, “For those types of structures not covered entirely, for new structural uses for established materials, for new materials, (…), the provisions given in this document can be applied, but with possible need for supplements”.

The authors specify in this paper parameters necessary for designing the basic load case of a wall structure made from medium-size elements, that is, the wall mainly under vertical loading. Characteristic and design compression strength was determined using the “design assisted by testing” method.

The cited papers paid attention to increased deformations of structures caused by mismatching, irregularities and different heights of structural elements, which are not levelled out by a layer of mortar in joints. Material models proposed in this paper for analysing structures, designing cross-sections, and estimating second-order effects on load-bearing capacity of walls considered those increased deformations. As a result, vertical strains (perpendicular to layers of the structure) could be determined more precisely, which is particularly significant for determining factors reducing load-bearing capacity at a mid-height of the wall. By knowing these strains, the behaviour of render and cladding elements related to the structure can be analysed. If required, a difference in strains of more and less loaded parts of the structure can be determined, as in the case of the area under the window opening and the connection area between load-bearing and non-bearing walls.

## 2. Materials, Structural Units, Specimens and Methods

### 2.1. Perlite

Perlite is a naturally occurring volcanic material and a type of aluminosilicate volcanic glass [50,51], that is, an amorphous product formed by fast cooling of lava or magma in the aquatic environment. Perlite comes from the term perlstein given by 19th century German petrologists for glassy rock with numerous concentric cracks, crushed fragments of which resembled pearls. An important feature of perlite is 2–5% water content. When heated quickly to a temperature of 760–900 °C, the bound water turns into steam and creates many bubbles, which increase volume by 10–20 times and provide perlite exceptional properties, that is, porous structure and low weight [52,53]. This type of material is known as expanded perlite. Perlite is mainly composed of SiO_2_ (70–75%), Al_2_O_3_ (12–18%), K_2_O, Na_2_O, and contains small amounts of Fe_2_O_3_, CaO, MgO, and TiO_2_ [54,55]. Perlite ores can be found all over the world. Large quantities of this material are extracted in Turkey, Greece, the USA, Japan, Italy, and Hungary [51,52,56].

There have been previous attempts to use other lightweight materials to construct masonry elements, not documented by experimental research results, including those made of autoclaved aerated concrete or using expanded clay aggregate. Structural units used for the construction of the tested dry masonry panel were made of material containing mineral binders and expanded perlite light aggregate. This is a new system of constructing dry masonry made available to the authors, which has not yet been subjected to experimental testing. The authors have not encountered the use of this type of aggregate in materials and structures whose function is to transfer loads. Expanded perlite has so far been used mainly as an insulating material, for example, plasters with low thermal transmittance. The use of expanded perlite significantly reduces the wall thermal conductivity coefficient. Low bulk density enables the production of blocks of relatively large sizes, which in turn allows for faster construction of walls and is associated with reduced labour costs. Other components and manufacturing technology are proprietary information of the manufacturer.

### 2.2. Structural Elements

**Figure 1:**
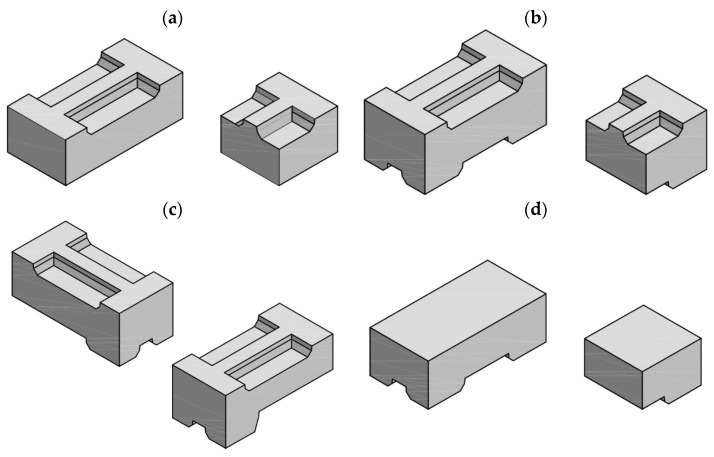
Structural units: (**a**) bottom, (**b**) stretcher, (**c**) corner, (**d**) top.

The presented system of erecting wall structures without mortar is composed of four types of medium-size elements—bottom elements S0 (Figure 1a), stretcher elements S1 (Figure 1b), top elements SZ (Figure 1c), and corner elements SN (Figure 1d). The fundamental nominal length of elements is 704 mm, and their nominal width is 352 mm. A total nominal height of stretcher and corner elements is 300 mm, and 250 mm for bottom and top elements. There are half elements with a length of 352 mm for the first three types of elements. Corner elements are available in the left and right version. All structural elements have specially profiled joints (Figure 1) to assemble wall structures without using mortar. The joints are “bidirectional”, which means that they better connect individual blocks in both planes perpendicular to the plane of the wall.

A manufacturer of the elements declared they were produced in conformity with the standard EN 771-3 [57]. Deviations in dimensions were declared for elements from D4 category, which means acceptable deviations in mm: +1; −3 for length and width, and ±1 for height. Flatness of bed faces and their parallelism were declared <1 mm.

The density class of elements declared by a manufacturer is 390 ± 10% kg/m^3^. The declared thermal conductivity factor is 0.084 ± 0.003 W/m·K.

The compressive strength of structural elements was determined in accordance with the standard EN 772-1 [58], which is relevant for testing masonry units. Specimens were prepared and cured as masonry units made of autoclaved aerated concrete, which is the most similar material in terms of composition, dimension ratio, density, and strength. Tests on compressive strength were conducted on 12 cubes with a side length of 100 mm, cut out from whole elements. Dixon and Grubbs tests demonstrated there was no basis for rejecting any outliers. Table 1 presents test results for structural elements of this material under compression. The Shapiro-Wilk test confirmed a hypothesis that a specimen originated from the population with a normal distribution. Hence, the mean interval compressive strength was determined for the population at the statistical significance 0.10.

Compressive strength f_B_ was not lower than 1.5 N/mm^2^ according to the manufacturer’s declaration. Determination of normalized strength for masonry units according to Annex A to the standard EN 772-1 [58] requires that moisture content and shape of specimens is included. The conversion factor for the air-dried state was 0.8 because the specimens were oven-dried. The conversion factor for cubes with a side length of 100 mm was equal to 1.0. Hence, the normalized compressive strength of structural elements f_b_ was equal to 1.32 N/mm^2^.

### 2.3. Panel Specimens and Methods

**Figure 2:**
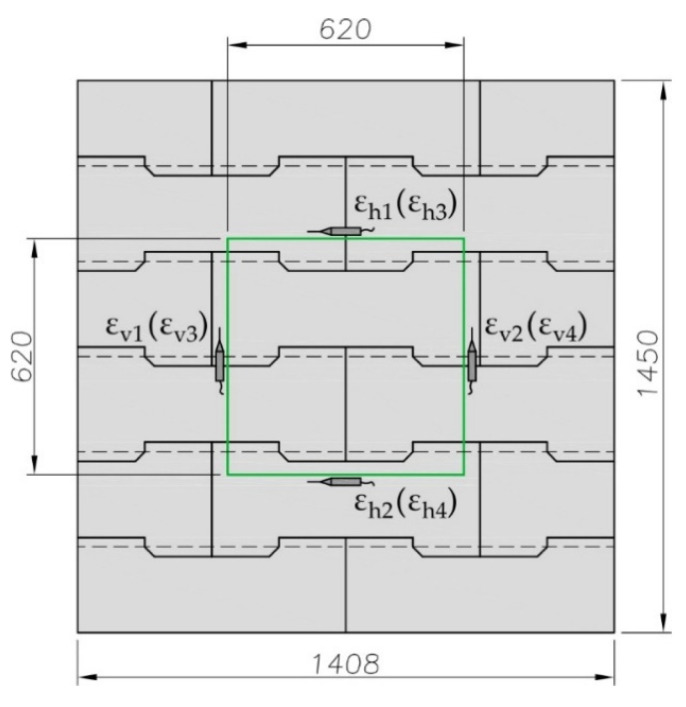
Panel specimen.

The compressive strength of the structure made of the structural elements under discussion was assumed to be determined by analogy to a masonry structure according to the standard EN 1052-1 [59]. Figure 2 illustrates the shape, structure and dimensions of the specimen conforming to the standard mentioned. The specimen was composed of six layers. Due to the presence of profiled joints, conventional horizontal surfaces of individual layers were marked with dashed lines in Figure 2. The conventional height of external top and bottom layers was 225 mm, while the height of internal layers was 250 mm. Structural elements of the system were used to build single-leaf walls with a width equal to the width of these elements (352 mm). Slenderness of specimens was 4.1.

The mean vertical ε_vi_ and horizontal ε_hi_ (i = 1, 2, 3, 4) strains of the specimen were determined on the basis of displacements of corner points forming a square measuring system with a side length of 620 mm (green lines in Figure 2). Displacements were measured on both sides of the specimen using transducers with an accuracy of 0.002 mm.

The specimens were subjected to monotonic loading at a ratio of 0.07 N/mm^2^·min until their failure, that is, the moment when there was no increase in load. The tests were performed on nine specimens.

**Figure 3:**
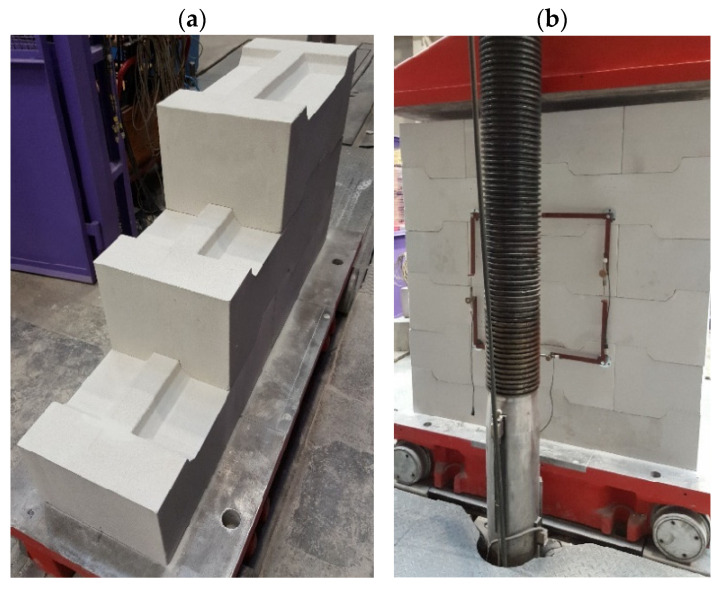
Specimens: (**a**) during erection, (**b**) prepared for testing.

Figure 3 illustrates specimens during assembly and positioning in the testing machine.

## 3. Results

### 3.1. Mean Panel Compresive Strength

Table 2 presents basic test results for compressive strength. The resultant failure force P_ui_ and corresponding values of line load p_ui_ kN/m are shown in Table 2. Such information is practical as walls subjected to mainly vertical load usually transfer linear load.

The data obtained were intended to provide information required to design structures. Therefore, apart from slight dispersion of test results (ν_f_ = 4.07%), the quality of the results obtained was also important with reference to the normal distribution of the parameter examined in the population. The distribution of compressive strength did not significantly vary from the normal distribution. This is confirmed by a minor difference between the mean value and the median (0.6%), a slight right-side skewness and slightly platykurtic distribution. It is assumed that absolute values of skewness lower than 1 and kurtosis lower than 2 entitle us to conduct parametric statistical tests. The Shapiro-Wilk test confirmed a hypothesis that specimens originated from the population with a normal distribution. Dixon and Grubbs tests demonstrated that there was no basis for rejecting any outliers. The mean interval value and the interval value of the standard deviation could be also determined with the assumed probability.

The compressive strength of the panels was, as expected, lower than the strength of the block material. Due to the low compressive strength of the tested dry masonry, it is expected that the use of this type of material for load-bearing walls will be limited mainly to low-rise buildings with low loads, mainly single-family houses, residential and office buildings. This system could also be used for the construction of single-storey buildings with higher wall heights and a light roof structure, e.g., sports or commercial buildings.

The ratio between mean strength of specimens and compressive strength of structural elements was f/f_B_ = 0.65 (see Table 1 and Table 2). The studies presented in [25] achieved a ratio of approx. 0.50, while the tests were performed on a dry stack composed of two structural elements. The tests presented in [26] achieved a ratio f/fB = 0.39, whereas the tests described in [27] achieved a ratio f/f_B_ = 0.40–0.20. The greater the strength of structural elements f_B_, the lower the ratio f/f_B_.

### 3.2. σ-ε Relationship

**Figure 4:**
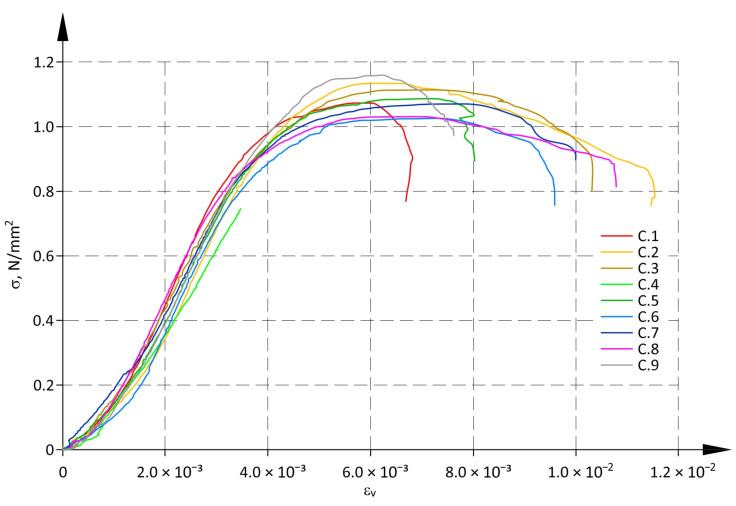
σ-ε relationship for vertical strains.

**Figure 5:**
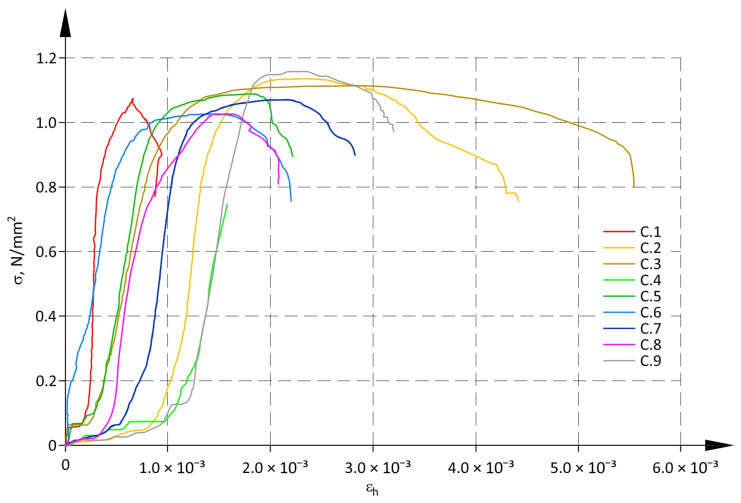
σ-ε relationship for horizontal strains.

Figure 4 shows diagrams of σ-ε_v_ relationships obtained from tests on all specimens. Figure 5 illustrates relationships between stress and mean horizontal strains σ-ε_h_. Due to the failure of measuring instruments, the reading of displacement for one specimen (C.4) was taken to a value equivalent to 70% of the maximum load. In that case, the ultimate force was read from the testing machine.

Both in the case of vertical ε_v_ and horizontal ε_h_ strains, significant increases in strains were noticed at relatively small values of stress within 0–0.2f range. Relationships σ-ε were not linear in that range. A similar effect, but under tensile stress, was found during tests on composite materials and in biomechanics [60]; similar behaviour was exhibited by tissues in living organisms, e.g., collagen [61]. Such a non-linearity range is usually known as the toe region or the toe and heel regions. For materials composed of fibres, the toe and heel regions were a consequence of straightening of wired fibres or fibres arranged in a direction different to the direction of tensile load.

The toe region observed for the compressed dry stack panels is the effect of structural element imperfections and mismatch of joints with values within a range of tolerances declared by the manufacturers. Low compressive stress resulted in closing gaps between the elements, which consequently increased vertical strains. This effect is not observed in masonry structures as mortar is used to join masonry units. Mortar acts as the “cushion”, which minimizes the concentration of stresses in contact areas between the masonry components and eliminates irregularities on the surface of units.

The occurrence of increased deformations at low values of compressive stress (compression toe) was expected after the analysis of dry masonry tests published so far and mentioned in the introduction. From a practical point of view, larger wall deformations may be important in the case of adjacent walls with significantly different stresses. Such differences are usually the cause of cracking. It should also be remembered that deformations of load-bearing compressed elements affect second-order effects and, consequently, the load-bearing capacity of the walls. This effect is usually considered using coefficients that reduce the load-bearing capacity.

In the panel subjected to mainly vertical load (perpendicular to the panel layers), horizontal strains ε_h_ (parallel to the panel layers) were caused by tensile stress. In the above situation, horizontal tensile stress could loosen vertical or inclined joints between dry stack structural elements, and their closure was also possible. Consequently, the behaviour of the structure could lead to significant horizontal strains under small loads. A dispersion of ε_h_ values in Figure 5 confirmed the described behaviour. There was almost a 15-fold difference between minimum and maximum strains ε_h_ under vertical stress of 0.2f (0.217 N/mm^2^). The coefficient of variation ε_h_ for all specimens at σ = 0.2f exceeded 60%.

It was observed that the ratio of strains ε_h_ at σ = 0.2f and strains under maximum stress σ = f was considerably larger than for vertical strains ε_v_. As a result, the diagrams of σ-ε_h_ relationships between the toe region and the area of plastic strains were almost vertical.

For both relationships σ-ε_v_ and σ-ε_h_ three intervals could be identified: two non-linear sections for the toe region and plastic strains, and one near-linear interval between non-linear sections.

### 3.3. Cracking

**Figure 6:**
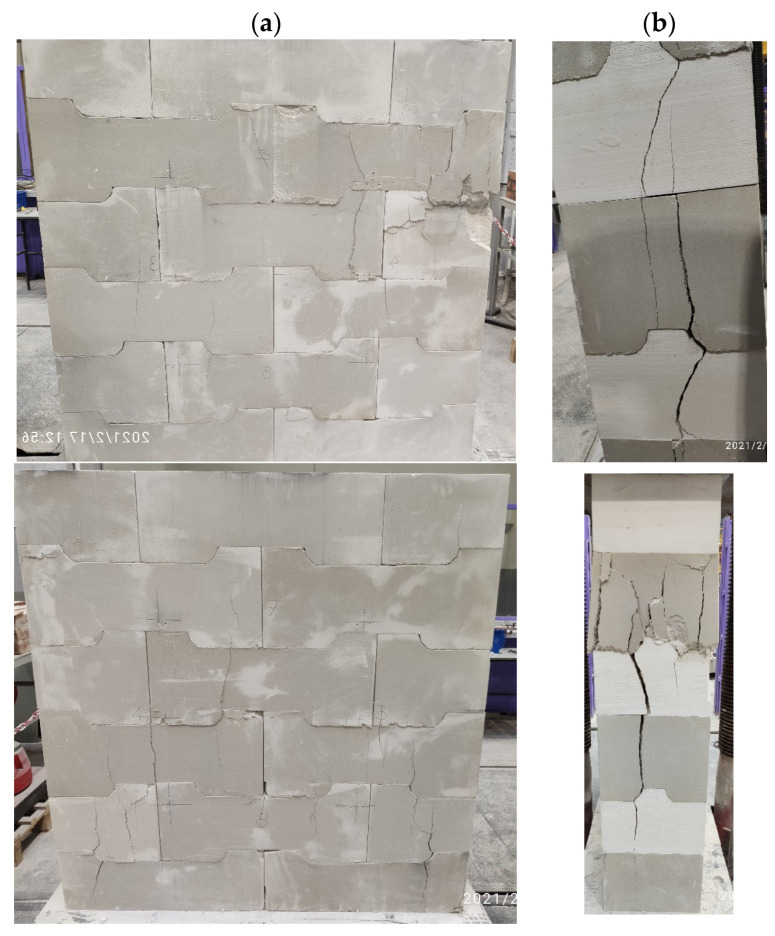
Cracking pattern (**a**) on lateral sides of the specimens, (**b**) inside the panels.

Noticeable cracks on specimens were initiated under a load resulting in compressive stress within a range of 0.4–0.6 N/mm^2^, that is, under a stress lower than the design compressive strength (see Table 3). First cracks on side surfaces of the specimens usually started to propagate in places of concentrated stresses, that is, at the ends of structural elements or in the corners of the interlocking joints (see Figure 6a). Cracks running from joint corners also appeared inside the specimens, that is, along planes not seen in case of real wall structures (see Figure 6b).

## 4. Analysis and Discussion of Test Results

Safe design of structures requires knowledge of characteristic and design values of material strength. The published results of the studies cited in the introduction [24,25,26,27,28,29,30,31,32,33,34,35,36,37,38,39,40,41,42,43,44,45,46,47,48] present the results of experimental studies and theoretical analyses. Usually, however, the authors of these studies do not provide information on mechanical parameters that could be used directly in design practice. Analysis of the behaviour of a structure under load requires at least knowledge of the σ-ε relationship. The standards for designing structures provide the simplest possible models of material behaviour. In the case of brittle materials such as concrete or masonry, the engineer can usually use a parabolic, parabolic-rectangular or linear model. Similarly, in the case of models used for designing cross-sections, in addition to more complex relationships, the stress distribution in the compressed zone of the cross-section can be replaced by an equivalent rectangular distribution. Some studies published in the literature show experimentally obtained σ-ε relationships [24,25,32,33,34,39], but none of them propose a mathematical model that can be used in practice.

The analysis of test results described in this paper was aimed at providing information required to conduct linear and non-linear analyses of structures, including the proposed linear and non-linear material models. From an engineer’s point of view, it is also important to have information necessary for choosing the material model for computing deformations of a structure and safe designing of cross-sections.

In the tests of panels made of blocks with perlite aggregate described in this article, the mean compressive strength of 1.085 N/mm^2^ was obtained at the mean deformation of 6.81 × 10^−3^. In the available publications presenting the results of compressive strength tests under axial load, the authors determine the maximum stress for the specimens and do not always specify the accompanying deformations. The researchers did not determine the characteristic and design strengths:

- The research published in [18] concerned a dry masonry made of lightweight foam concrete blocks with a bulk density of 1250 kg/m^3^. The mean value of the prism strength was 1.30 N/mm^2^.
- High dry masonry panels 3.0 m wide and 2.5 m were tested in [27]. The panels were made of soil and cement units formed using the hydraform method under a pressure of 10 N/mm^2^. The compressive strengths of the panels ranged from 1.98 to 4.53 N/mm^2^ and depended on the compressive strength of the masonry units. The units had a mean compressive strength of 5 to 23 N/mm^2^, depending on the amount of cement.

The following works described tests on dry masonry made of concrete blocks:

- In [26], tests of three-layer prisms were presented. The mean compressive strength obtained was equal to 11.2 N/mm^2^, while the mean strength of concrete blocks was equal to 23.4 N/mm^2^.
- Tests of prisms were published in [25]. The obtained compressive strengths of the prisms ranged from about 7.0 to over 15.0 N/mm^2^, depending on the strength of the concrete blocks, which was between 15.5 to 25.4 N/mm^2^. The strains associated with the maximum stress ranged from about 4.2 × 10^−3^ to approximately 5.4 × 10^−3^.
- In [32], the results of tests of prisms consisting of three masonry elements of different lengths were published. The average compressive strengths ranged from 28.0 N/mm^2^ (for the prisms of the longest length—445 mm) to 35.0 N/mm^2^ (for the shortest prisms—145 mm). The authors did not specify the strength of the concrete blocks. The deformations obtained at the maximum compressive stress ranged from 1.8 × 10^−3^ to 2.1 × 10^−3^.

It should be noted, however, that in the cited publications the tests were performed on specimens other than those specified in the EN 1052-1 standard, including prisms, therefore a direct comparison of these results is not reliable.

### 4.1. Characteristic and Design Compressive Strength

Characteristic and design compressive strengths were determined from test results according to requirements specified in Annex D “Design assisted by testing” to the standard EN 1990 [62]. However, this method could be used only if the distribution of an analysed variable is normal or log-normal. This condition was met in case of the distribution of compressive strength values (see Section 3.1).

The procedure specified in the standard EN 1990 assumed that the mean value m_x_ determined on the basis of the previous tests was unknown, and the coefficient of variation could be “known” or “unknown”. The “known” value could be interpreted as the value ν_x_ determined by good engineering judgement and professional expertise, which cannot be justified by mathematical considerations alone due to the lack of information on previously determined mean value and standard deviation [63]. In case of the tests discussed, the coefficient ν_x_ was considered to be “unknown” and should be determined from the results obtained for the specimens, as in case of the mean and the standard deviation. However, in the standard EN 1990 there is a provision that states if ν_x_ is “unknown”, then values lower than 10% should not be taken for calculations. The coefficient of variation determined during the tests barely exceeded 4% (see Table 1). The least favourable value of this coefficient determined with a 90% probability from extreme interval values of standard deviation and the mean value of the population slightly exceeded 7%. Therefore, the conservative characteristic compressive strength was determined according to the standard assuming that the coefficient of variation ν_x_ is equal to 10%:

(1) $$f_{k}=f\left(1-\nu_{x}k_{n}\right)=1.085\cdot \left(1-0.1\cdot 1.96\right)=0.873\;N/{\mathrm{mm}}^{2},$$

where f means compressive strength (see Table 2), and k_n_ is a sample size-dependent coefficient.

The design strength in the Limit States Method is determined by dividing the characteristic strength by the partial safety factor for materials γ_m_. The safety factor γ_m_ was unknown for the material analysed. Therefore, the design strength was again determined according to provisions specified in standard EN 1990. The design strength was determined from the equation similar to the relationship (1), where the value of coefficient k_d,n_ depended on the number of analysed specimens.

(2) $$f_{k}=f\left(1-\nu_{x}k_{d,n}\right)=1.085\cdot \left(1-0.1\cdot 4.79\right)=0.565\;N/{\mathrm{mm}}^{2}.$$

Determined values of compressive strengths and the corresponding values of mean vertical strains are shown in Table 3. The coefficient of variation for strains is given in brackets. As there was a significant spread of values for horizontal strain ε_h_ (see Figure 5), they were not included in the further analysis, and the symbol ε was used only for vertical strains.

The ratio of the characteristic strength to the design strength f_k_/f_d_ was 1.55. Annex A to the current European standard for designing masonry structures EN 1996-1-1 [49] allows for a partial factor for materials γ_m_ equal to 1.5.

**Figure 7:**
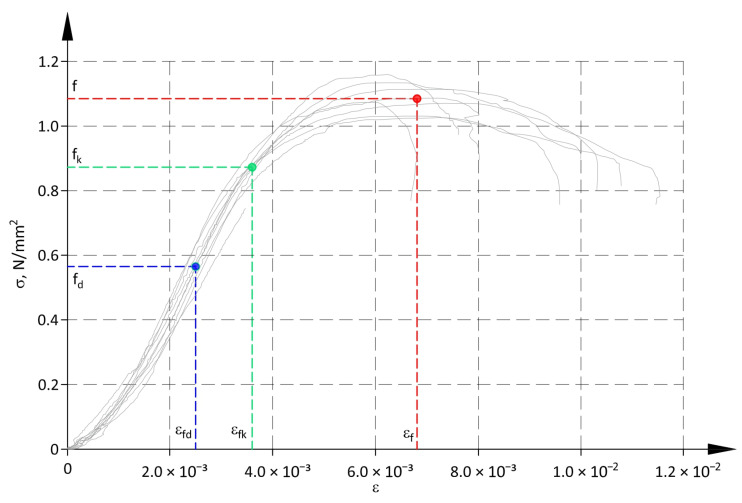
Compressive strengths of panels and corresponding strains.

A graphical presentation of determined strength values against the relationship σ-ε is shown in Figure 7.

### 4.2. Deformability

**Figure 8:**
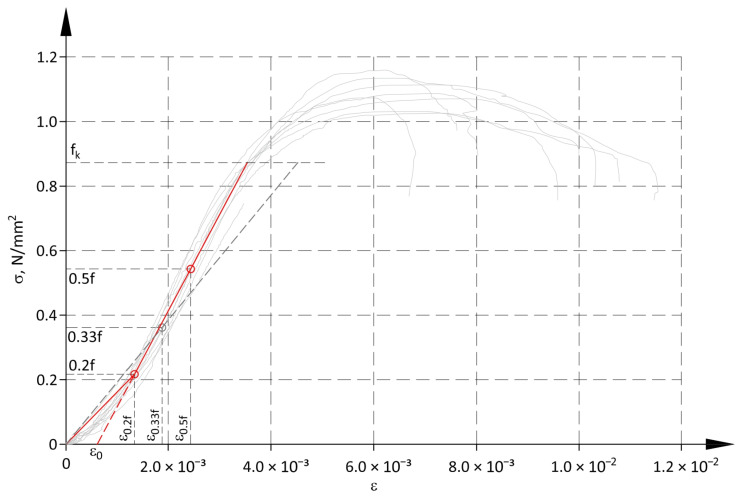
Method for determining the modulus of deformation.

In the case of masonry structures, the modulus of longitudinal elasticity was determined according to the standard EN 1052-1 [59] from two points of the σ-ε relationship, that is, the beginning of the coordinate system and the point with coordinates equal to 1/3 of the mean compressive strength and the corresponding mean vertical strains. A dashed grey line in Figure 8 runs through the points mentioned. However, this straight line does not reflect the stress-strain relationship.

Considering the obtained relationship σ-ε, two values of the modulus of deformability were determined for different ranges of the σ-ε relationship. The linear approximation of non-linear relationship was assumed to be in the first interval, which means that this relationship includes the toe region. The range of this interval was assumed from the origin of the coordinate system to the point specified by the stress equal to 20% of the mean compressive strength and the corresponding mean strain ε_0.2f_. The second interval was assumed to be valid within a range of stresses from 0.2f to f_k_. The straight-line model in the second interval runs through points with coordinates 0.2f and ε_0.2f_, and 0.5f and ε_0.5f_. The real relationship σ-ε obtained from the tests for a range of stresses from 0.2f to 0.5f was an almost perfectly linear relationship, which is visible in Figure 8. For the stress value close to the strength f_k_, the σ-ε relationship obtained from tests only slightly deviated from a straight line. Table 4 presents parameters of the deformability model for the panels analysed and values of the modulus of deformability. The authors intentionally did not use the term “modulus of elasticity” and the commonly used symbol “E”, as strains in the first interval were not elastic.

The mathematical model describing the total deformations of the structures analysed can be expressed with the following equations:

(3) $$\varepsilon =\frac{\sigma }{D_{1}}\;\mathrm{for}\;0\leq \sigma <0.2f,\;\varepsilon =\frac{\sigma +\left(D_{2}-D_{1}\right)\varepsilon_{0.2f}}{D_{2}}\;\mathrm{for}\;0.2f\leq \sigma \leq f_{k}.$$

The significant spread of values of horizontal strains (see Section 3.2 and Figure 5) did not allow for determining the reliable value of Poisson’s ratio.

### 4.3. Material Models for Structure Analysis

The authors propose to consider two material models specified on the basis of the test results. Both models were composed of three parts.

**Figure 9:**
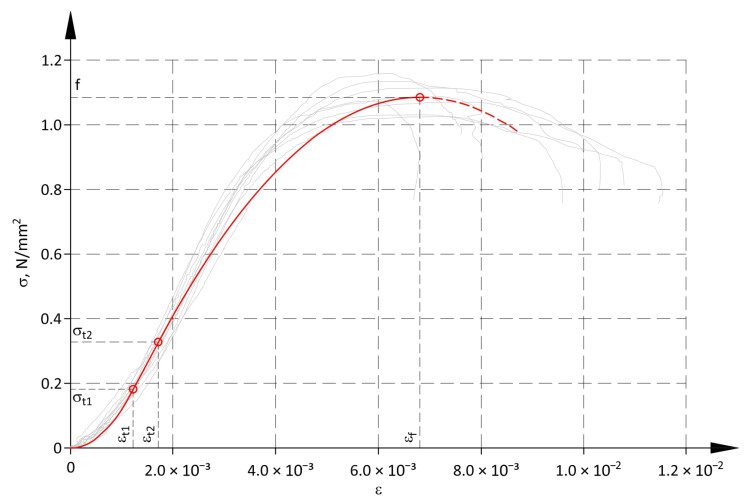
Non-linear model for analysis of structure.

The first model, which is more complex but represents more adequately the real σ-ε relationship, is shown in (Figure 9). The first branch is a parabola in the toe region within a range of stresses up to σ_t1_. The second branch is linear between points determined by stresses σ_t1_ and σ_t2_ and the corresponding strains ε_t1_ and ε_t2_. The linear part is compatible with the relationship shown in Figure 8 and Table 4, and determined from the points (ε_0.2f_, 0.2f) and (ε_0.5f_, 0.5f). The third branch is parabolic and ran between the points with coordinates (ε_t2_, σ_t2_) and (ε_f_, f). Both parabolic branches are tangent to the linear part at points (ε_t1_, σ_t1_) and (ε_t2_, σ_t2_). This construction of the non-linear model results from the fact that it should be as simple as possible, and it is not possible to use one third-degree curve or two tangent parabolas. It is the simplest solution with reference to the mathematical description, which considered that those curves had to run through the origin of the coordinate system and the point (ε_f_, f), and simultaneously had to represent the real path of the σ-ε relationship. The quality of the non-linear model fit to the results of the panel tests was determined by the Pearson correlation coefficient r. For the first branch, r = 0.876 was obtained, for the linear branch r = 0.707 and for the parabolic third branch r = 0.955. Parameters of the non-linear model are presented in Table 5.

The non-linear model can be described with the following equations:

(4) $$\sigma =k_{1}\varepsilon^{2}\;\mathrm{for}\;0\leq \varepsilon <\varepsilon_{t1},\;\sigma =D_{2}\left(\varepsilon -\varepsilon_{t1}\right)+\sigma_{t1}\;\mathrm{for}\;\varepsilon_{1t}\leq \varepsilon <\varepsilon_{2t},\;\sigma =k_{2}{\left(\varepsilon_{f}-\varepsilon \right)}^{2}+f\;\mathrm{for}\;\varepsilon_{2t}\leq \varepsilon \leq \varepsilon_{f}.$$

**Figure 10:**
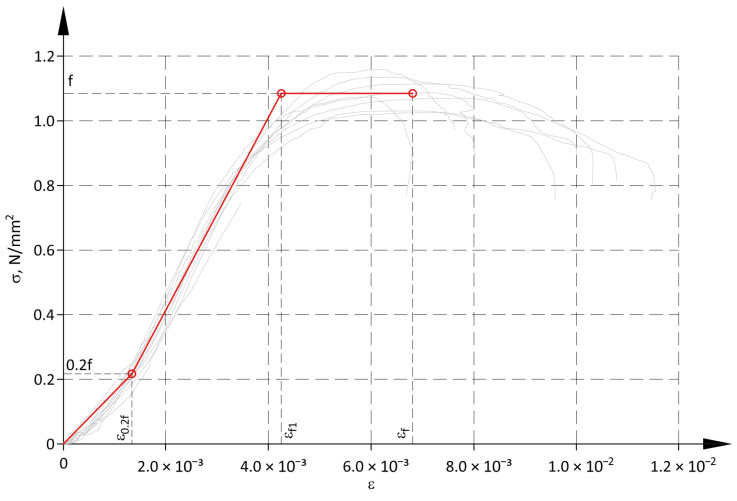
Linear model for analysis of structure.

The second proposed model for the structure analysis is more simplified, and consequently less precise. It is a tri-linear model (Figure 10). The course of the first two lines is consistent with the model used to determine the modulus of deformability (see Figure 8 and Table 4). However, the top boundary of the second branch reached the mean compressive strength f. At that point, the strain reached the value ε_f1_. The third branch described plastic strains within a range from ε_f1_ to ε_f_, under constant stress f. Parameters used to develop the linear model are shown in Table 6.

The linear model is described with the following Equations:

(5) $$\sigma =D_{1}\varepsilon \;\mathrm{for}\;0\leq \varepsilon <\varepsilon_{0.2f},\;\sigma =D_{2}\varepsilon -\left(D_{2}-D_{1}\right)\varepsilon_{0.2f}\;\mathrm{for}\;\varepsilon_{0.2f}\leq \varepsilon <\varepsilon_{f1},\;\sigma =f\;\mathrm{for}\;\varepsilon_{f1}\leq \varepsilon \leq \varepsilon_{f}.$$

### 4.4. Material Models for Cross-Section Design

**Figure 11:**
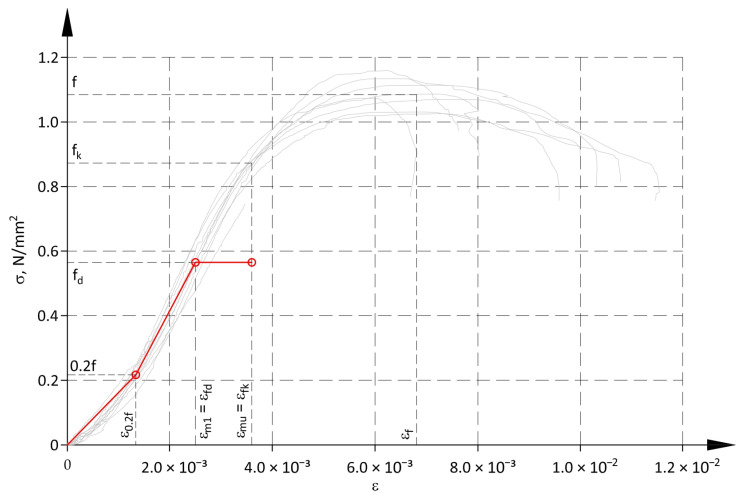
Material model for cross-section design.

The material model for cross-section design was assumed to be tri-linear, taking into account larger deformations of structures under stress within a range of 0–0.2f (Figure 11). The first branch of the model runs from the origin of the coordinate system to the point with coordinates ε_0.2f_, 0.2f (Table 4). The end of the second line is at the point where stress reaches the design compressive strength f_d_ and strain ε_m1_, the value of which is assumed to be equal to the strain ε_fd_ (Table 3). The third branch of the model represents plastic strains within a range from ε_m1_ to ε_mu_. Strains ε_m1_ and ε_mu_ are mean values determined under the stress equal to respectively the design f_d_ and characteristic f_k_ compressive strength. The ratio of ε_mu_/ε_m1_ is 1.44 and is within the range of 1.14–1.75 specified in Eurocode 6 [49] and is typical for deformations of walls made of masonry units from Group 1.

**Figure 12:**
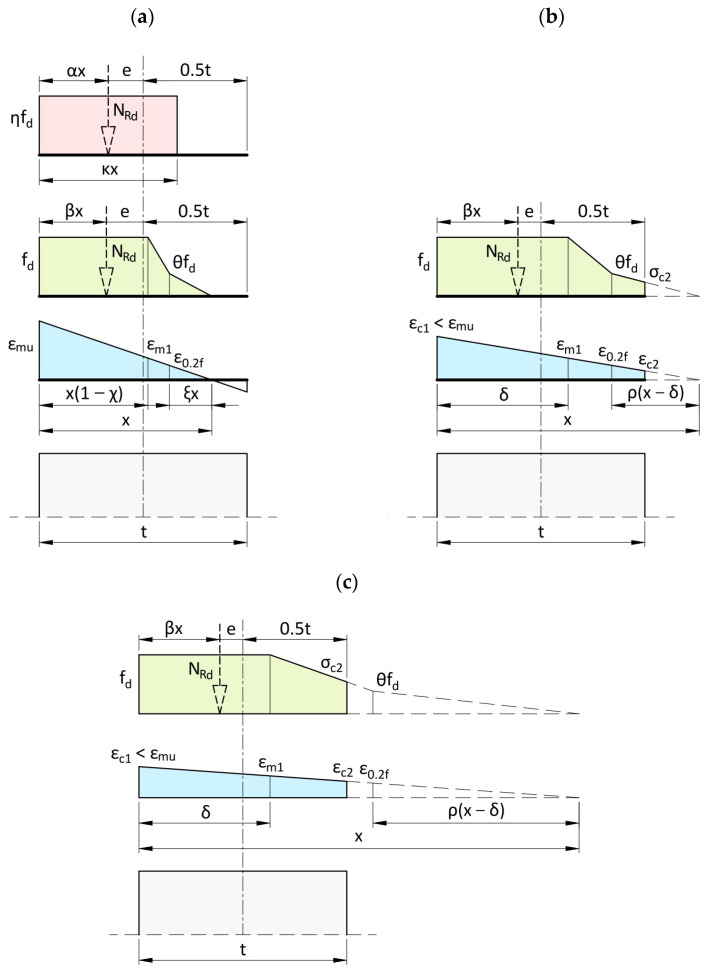
Distribution of stresses and strains in the compressed cross-section where (**a**) x ≤ t, (**b**) x > t and ε_c2_ < ε_0.2f_, (**c**) x > t and ε_c2_ ≥ ε_0.2f_.

Table 7 presents parameters of the tri-linear model, and their meaning is illustrated in Figure 12. Parameters of the model were defined for three ranges: at x ≤ t and x > t depending on strain values of the less compressed edge of the cross-section ε_c2_ < ε_0.2f_ or ε_c2_ ≥ ε_0.2f_. For the cross-section nominally under axial compression (e = 0), strain should not exceed the value ε_m1_. It also means that assuming the principle of plane cross-sections according to Euler-Bernoulli theory, strain values cannot exceed ε_m1_ within a distance greater than δ from the more compressed edge of the cross-section (Table 7, Figure 12).

The equivalent rectangular distribution of compressive stresses can be also applied for designing cross-sections of structures of this type. Parameters for such a model were determined assuming that the stress block in the equivalent model is equal to the area of the stress block in the tri-linear model. Equality of these areas could be provided using the coefficient κ reducing the height of the cross-section under compression, the coefficient η reducing allowable compressive stress, or applying both of them (Figure 12a). Additionally, to obtain similar values of load-bearing capacity of the cross-section N_Rd_ for the tri-linear and rectangular models, the distance between the resultant force and the edge of the cross-section should be approximately the same. Hence, the applied parameters α and β should be as equal as possible (Figure 12a).

**Figure 13:**
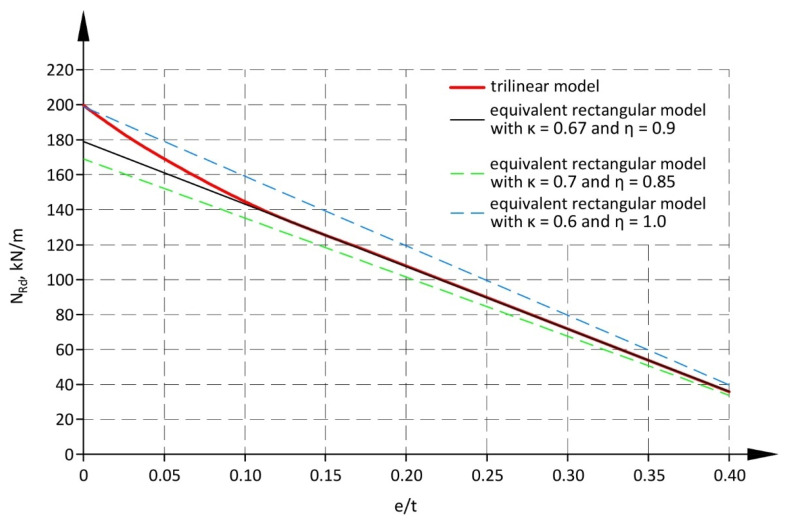
Comparison of models for cross-section designing—a tri-linear model and different types of the equivalent rectangular distribution.

Figure 13 illustrates the diagram of values of load-bearing capacity of the compressed cross-section (without second-order effects) obtained for the tri-linear model and different types of the equivalent rectangular distribution. Parameters of the rectangular distribution κ = 0.67 and η = 0.9 provided nearly the same load-bearing capacity and conservative estimation at e/t < 0.10. Table 8 presents parameters proposed for the equivalent rectangular distribution of compressive stress.

### 4.5. Effect of Eccentricity of Loading on Capacity of the Cross-Section Under Compression

Load eccentricities of masonry walls, where compressive stresses are usually caused by gravity-type loads (walls subjected mainly to vertical load) were determined for cross-sections at the top edge of the wall (below the floor of the storey above), at the bottom edge of the wall (above the floor of the storey below or above the foundation), and at a mid-height of the wall. The first-order eccentricity e, which is usually a sum of the eccentricity of vertical load e_i_, the eccentricity e_h_ resulting from the horizontal load, e.g., wind load, and the initial eccentricity e_init_, were determined for all these cross-sections. In accordance with guidelines of Eurocode 6 [49], the eccentricity e_init_ is considered as equal to 1/450 of the effective height of the wall.

In cross-sections at the top and bottom ends of the wall, the design load-bearing capacity of the compressed cross-section N_Rd_, including the total eccentricity e, could be determined from equations specified in Table 7 for the tri-linear model, and in Table 8 for the equivalent rectangular distribution of stresses.

In the previous version of Eurocode 6 from 2005, the eccentricity e_k_ resulting from masonry creeping was determined for the cross-section at the mid-height of the wall. The current version of the standard does not include e_k_ eccentricity.

The cross-section at the mid-height of the wall should include deformations of the wall related to the second-order effects and an increase in the load eccentricity. The coefficient of reduction is one of the methods that take into account the additional eccentricity

(6) $$\Phi_{m}=\frac{N_{\mathrm{Rd}}}{N_{R0}}=\frac{N_{\mathrm{Rd}}}{f_{d}t},$$

where forces N_R0_ and N_Rd_ are design load-bearing capacity of one meter of the wall.

The current version of the European standard for designing masonry structures [49] also presents the approach in which coefficient Φ_m_ is determined as a function of the wall slenderness:

(7) $$\lambda =\lambda_{1}\sqrt{\frac{f_{k}}{E}}=\frac{h_{\mathrm{ef}}}{t}\sqrt{\frac{f_{k}}{E}}.$$

The phenomenon of failure of compressed bars is usually considered in terms of the material’s destruction, and failure as a result of loss of stability. Slenderness λ_t_ distinguished those two cases. For a wall slenderness smaller than λ_t_, the factor Φ_m_ specifying material failure can be determined with Johnson’s parabola [64] in a general form expressed as follows:

(8) $$\Phi_{m}=\Phi -{C\lambda }^{2},$$

where Φ is the coefficient of reduction determined depending on the eccentricity e, excluding the second-order effects.

Force causing loss of stability when λ ≥ λ_t_ can be determined with the coefficient Φ_m_ obtained from the following hyperbole equation:

(9) $$\Phi_{m}=\frac{A}{\lambda^{2}}.$$

Slenderness λ_t_ at the point of tangency of both curves, defined by Equations (8) and (9), is determined from the following equation:

(10) $$\lambda_{t}=\sqrt{\frac{2A}{\Phi }}.$$

Parameter A according to Angervo [65,66] for the linear model can be determined from the following equation:

(11) $$A=0.7875A_{1}^{3}=0.7875{\left(1-2\frac{e}{t}\right)}^{3}.$$

The value of the C parameter is defined as follows:

(12) $$C=\frac{\Phi^{2}}{4A}.$$

Thus, the factor Φ_m_ can be determined from [67] in the following way:

(13) $$\Phi_{m}=\left\{\begin{array}{cc} \Phi \left(1-\frac{\Phi }{4A}\lambda^{2}\right) & \mathrm{for}\;\lambda <\lambda_{t},\\ \frac{A}{\lambda^{2}} & \mathrm{for}\;\lambda \geq \lambda_{t}. \end{array}\right.$$

Eurocode 6 adopts this approach to determine Φ_m_ and, taking the rectangular distribution of compressive stresses, the coefficient of reduction is expressed with the following equations:

(14) $$\Phi =A_{1}=1-2\frac{e}{t},$$

(15) $$\Phi_{m.\mathrm{EC}}=\left\{\begin{array}{cc} A_{1}-\frac{\lambda^{2}}{2.58A_{1}} & \mathrm{for}\;\lambda <1.14A_{1},\\ 0.65\frac{A_{1}^{3}}{\lambda^{2}} & \mathrm{for}\;\lambda \geq 1.14A_{1}. \end{array}\right.$$

For the non-linear material model, the factor Φ against the first-order eccentricity e can be determined from the following relationships:

(16) $$\Phi_{\mathrm{nlin}}=\left\{\begin{array}{cc} \frac{\Phi_{\mathrm{cr}}}{\Phi_{\mathrm{cr}}+\left(1-\Phi_{\mathrm{cr}}\right)\frac{e}{e_{\mathrm{cr}}}} & \mathrm{for}\;\frac{e}{t}<\frac{e_{\mathrm{cr}}}{t},\\ \Phi_{\mathrm{cr}}\frac{1-2\frac{e}{t}}{1-2\frac{e_{\mathrm{cr}}}{t}} & \mathrm{for}\;\frac{e}{t}\geq \frac{e_{\mathrm{cr}}}{t}. \end{array}\right.$$

Eccentricity e_cr_ defines the limit position of compressive force, at which the cross-section is no longer fully compressed, and it is a constant value for a given material. Value e_cr_/t for the non-linear model, which does not have a falling branch after reaching the compressive strength f and does not include tensile strength, can be determined in accordance with [68,69,70] from the following equation:

(17) $$\frac{e_{\mathrm{cr}}}{t}=\frac{m_{\epsilon }\left(\epsilon_{u}=1\right)}{n_{\epsilon }\left(\epsilon_{u}=1\right)}-\frac{1}{2},$$

where

(18) $$n_{\epsilon }\left(\epsilon_{u}\right)=\int_{0}^{\epsilon }\bar{\sigma }d\varepsilon ,$$

(19) $$m_{\epsilon }\left(\epsilon_{u}\right)=\int_{0}^{\epsilon }\bar{\varepsilon }\;\bar{\sigma }d\varepsilon .$$

The non-linear model of the material can be expressed with the normalized relationship $\bar{\sigma }-\bar{\varepsilon }$. These values are standardized with reference to compressive strength of the wall f and the corresponding strain ε_f_. Relative strain ϵ_u_ is the ratio of strain ε_f_ to maximum strain ε_u_. For a non-linear material without a falling branch after reaching the maximum stress, ϵ_u_ = 1. The coefficient of reduction Φ_cr_, constant for a given material model, is determined [68] from the following equation:

(20) $$\Phi_{\mathrm{cr}}=\frac{1}{2}\frac{n_{\epsilon }{\left(1\right)}^{2}}{n_{\epsilon }\left(1\right)-m_{\epsilon }\left(1\right)}\left(1-2\frac{e_{\mathrm{cr}}}{t}\right).$$

**Figure 14:**
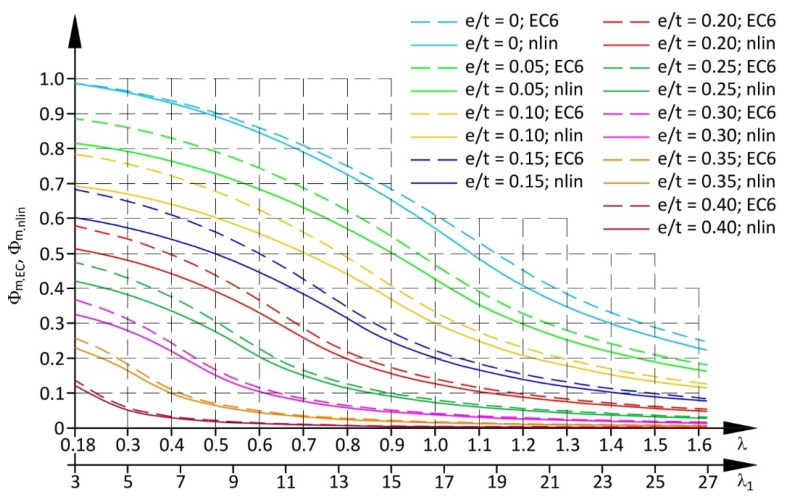
Comparison of reduction factors Φ_m.nlin_ (27) and Φ_m.EC_ (15).

Figure 14 illustrates diagrams of the coefficient Φ_m.EC_ determined in accordance with the approach specified by Eurocode 6 (14) and (15), considering increased strains of the structure at low values of compressive stress (dashed lines). The modulus of material elasticity determined in accordance with the standard EN 1052-1 [59] did not accurately specify the relationship between stress and strain determined from these tests (Figure 8—dashed grey line). Hence, slenderness of the wall considering greater strains at low compressive stress was determined from the following relationship:

(21) $$\lambda =\frac{h_{\mathrm{ef}}}{t}\sqrt{\frac{f_{k}}{D_{2}}+\varepsilon_{0}}=\frac{h_{\mathrm{ef}}}{t}\sqrt{\sim \varepsilon_{\mathrm{fk}}},$$

where D_2_ is the deformability parameter for the second linear branch of the model, and ε_0_ is the strain of value determined by the intersection point between this line and the horizontal axis of the diagram (Figure 8, Table 4).

The coefficient Φ_m.nlin_ was also determined for the non-linear model of the material analysed (Figure 9, Table 5) in accordance with Equations (11), (13), (16)–(21). Parameter A (11) was multiplied by the coefficient α, to account for the difference in deformations in the linear and non-linear model.

(22) $$A_{\mathrm{nlin}}=0.7875{\alpha A}_{1}^{3}=0.7875\alpha {\left(1-2\frac{e}{t}\right)}^{3}.$$

The factor α was defined as follows:

(23) $$\alpha =\frac{\varepsilon_{\mathrm{eq}}}{\varepsilon_{f1}}.$$

**Figure 15:**
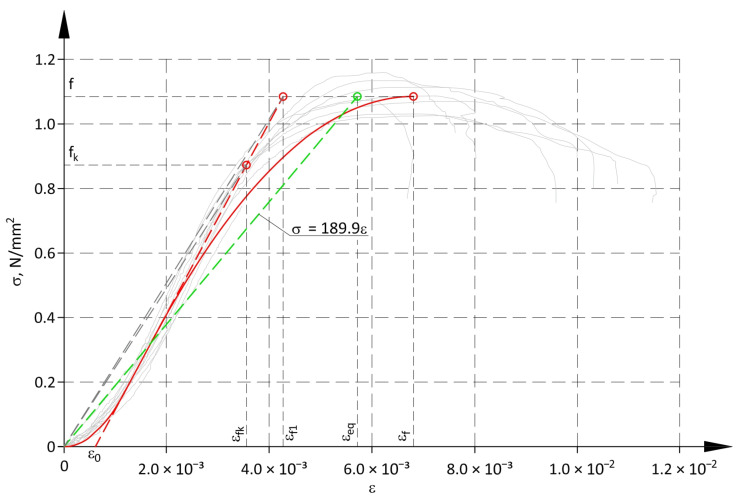
Method of determining strains for non-linear material model for determining the reduction coefficient Φ_m.nlin_ (non-linear material model—continuous red line; second part of the linear material model—dashed red line; linear approximation of the non-linear model—dashed green line).

Strain ε_f1_ was determined considering strains ε_0_ at relatively low compressive stresses (Figure 15).

(24) $$\varepsilon_{f1}=\frac{f}{D_{2}}+\varepsilon_{0}.$$

The strain ε_eq_ was determined from the directional coefficient of the linear equation (a dashed green line in Figure 15), which was used to approximate the non-linear relationship σ-ε with the method of least squares.

(25) $$\varepsilon_{\mathrm{eq}}=\frac{f}{189.9}.$$

Values of parameters required to determine the reduction coefficient for the applied non-linear model are shown in Table 9.

For the non-linear model, the reduction coefficient against the first-order eccentricity (in cross-section at the top and bottom edges of the wall) Φ_nlin_ for the dry stack wall analysed can be determined from the following equations:

(26) $$\Phi_{\mathrm{nlin}}=\left\{\begin{array}{cc} \frac{0.601}{0.601-0.399\frac{e}{e_{\mathrm{cr}}}} & \mathrm{for}\;\frac{e}{t}<0.160,\\ 0.884A_{1} & \mathrm{for}\;\frac{e}{t}\geq 0.160. \end{array}\right.$$

The coefficient Φ_m.nlin_ in the cross-section at the mid-height of the wall, including the first- and second-order effects, can be determined from the following equations:

(27) $$\Phi_{m.\mathrm{nlin}}=\left\{\begin{array}{cc} \Phi_{\mathrm{nlin}}\left(1-\frac{\Phi_{\mathrm{nlin}}}{2.35A_{1}^{3}}\lambda^{2}\right) & \mathrm{if}\;\lambda <1.08\sqrt{\frac{A_{1}^{3}}{\Phi_{\mathrm{nlin}}}},\\ 0.59\frac{A_{1}^{3}}{\lambda^{2}} & \mathrm{if}\;\lambda \geq 1.08\sqrt{\frac{A_{1}^{3}}{\Phi_{\mathrm{nlin}}}}. \end{array}\right.$$

A solid line in Figure 13 presents values of the coefficient Φ_m.nlin_ determined from Equation (27) for wall slenderness λ or λ_1_ = h_ef_/t and different relative eccentricities e/t.

Reduction coefficients calculated for the non-linear material model were smaller than those determined from the relationships specified in Eurocode 6. Differences were becoming greater as the slenderness of the wall was smaller, and the relative eccentricity of the load e/t was smaller (excluding the case where e/t = 0).

It should be emphasized that there are also other original methods for determining the deformability of the wall and the reduction coefficient for load-bearing capacity considering second-order effects for a wall of larger deformability. The papers by Knutsson [71,72,73], Jäger et al. [74], and Sandoval/Roca [75] can be mentioned in this context.

### 4.6. Desired Scope of Future Works

The above results can be used to analyse linear and non-linear structures and to design walls subjected to eccentric load, mainly vertical load.

Further tests on a dry stack wall made of blocks with perlite aggregate should specify other features that are required to design such structures. It can be assumed that these parameters are those already specified in standards for designing masonry structures, that is, in EN 1996-1-1 [49] and related standards.

Shear strength is an unknown parameter. Characteristic shear strength of the wall is usually limited due to tensile strength of the material. Eurocode 6 limits characteristic shear strength of the wall with unfilled head joints to 4.5% of the normalized compressive strength of masonry units f_b_. If the standard criteria are applied, then the characteristic shear strength f_vk_, in the case of the material described in this article, should not exceed the limit value f_vlt_ = 0.06 N/mm^2^. However, there is no adhesion in connection planes between structural units of dry masonry. In this situation, initial shear strength f_v0_ (at zero or minimum compressive stress perpendicular to layers of the wall) will therefore be associated with the failure of the joints profiled in structural units, which means that the value f_v0_ could be relatively high. Thus, it can be interesting to confront values f_v0_ and f_vlt_, at an unusual mechanism of shear failure with reference to masonry structures joined with mortar.

Another unspecified material parameter is flexural strength. In accordance with Eurocode 6, masonry structures have a characteristic flexural strength at failure in the plane perpendicular to bed joints f_xk1_ and a characteristic flexural strength f_xk2_ when the failure plane is perpendicular to bed joints. In case of the discussed dry stack wall, there is no flexural strength f_xk1_ due to a lack of adhesion forces in the planes of horizontal joints (no tensile strength in a direction perpendicular to these planes). In this situation, the design apparent flexural strength f_xd1,app_, could be applied. This term was defined in Eurocode 6 and it is a sum of strength f_xd1_ and design stress perpendicular to the plane of horizontal joints σ_d_. Thus, in the case of the structure discussed it can be assumed that f_xd1_ = σ_d_.

Wide-ranging tests are required for experimental verification of the reduction coefficient Φ_m.nlin_ shown in Figure 14 and given by Formula (27).

Shear modulus G is likely to be determined from testing diagonal tensile strength.

Also, the coefficient of thermal expansion and rheological parameters, including final creep coefficient and values of long-term moisture expansion or shrinkage, should be determined.

## 5. Concluding Remarks

This paper presents results of tests and analyses aimed at determining parameters for designing dry masonry structures made of medium-size blocks with expanded perlite aggregate, subjected mainly to vertical loads. The results of the tests included the following:

- the average compressive strength of dry masonry was determined as f = 1.085 N/mm^2^;
- the characteristic compressive strength f_k_ = 0.873 N/mm^2^ and the design strength f_d_ = 0.565 N/mm^2^ were estimated using the design assisted by testing method;
- in connection with the obtained strengths, it was suggested to use this type of material for the construction of low-rise buildings with low static loads, such as single-family houses, residential and office buildings or single-storey buildings with a light roof structure for sports or commercial purposes;
- for small compressive stresses, increased deformations of the structure were observed due to imperfections in the fit of the blocks (compression toe);
- two values of the deformability modulus were determined: in the compression toe area D_1_ = 162 N/mm^2^ and for the linear branch of the σ-ε relationship D_2_ = 297 N/mm^2^;
- it was found that it is impossible to reliably determine horizontal deformations under load perpendicular to the masonry layers, and thus also determine the Poisson’s ratio;
- non-linear and linear material models for the analysis of structures considering compression toe were proposed;
- a tri-linear model for the design of cross-sections of eccentrically compressed structures considering the increased deformations and an equivalent model with a rectangular block of compressive stresses were proposed;
- equations were defined for determining the coefficient Φ_m_ reducing the load-bearing capacity of eccentrically compressed elements due to second-order effects, considering the increased deformations in the compression toe;
- it was established that the determined coefficients Φ_m_ have lower values than those calculated, according to the Eurocode 6 standard for the design of masonry structures.

Comprehensive design of structures using the system described in the article requires further research. It is necessary to determine the characteristic and design values of shear and flexural strength and to determine the load-bearing capacity of the dry masonry under concentrated load. Parameters that allow for in-depth design also include final creep coefficient, moisture expansion or shrinkage, and coefficient of thermal expansion. A separate group of important findings are construction conditions, for example the connection of walls to roofs and floors.

## Figures and Tables

**Table 1:** Mean compressive strength of structural elements.

Mean Compressive Strength f_B_, N/mm^2^	Standard Deviation s_fB_, N/mm^2^	Coefficient of Variation ν_fB_, %	Confidence Interval with 90% Probability, N/mm^2^
1.65	0.146	8.87	1.57–1.72

**Table 2:** Test results for compressive strength of specimens.

Specimen	Ultimate Load		Compressive Strength f_i_, N/mm^2^	Mean Compressive Strength f, N/mm^2^		Standard Deviation s_f_, N/mm^2^	Coefficient of Variation ν_f_, %
	P_ui_, kN	p_ui_, kN/m					
C.1	531.6	377.6	1.073	1.085		0.0441	4.07
C.2	562.0	399.1	1.134				
C.3	551.6	391.8	1.113				
C.4	535.0	380.0	1.079				
C.5	538.5	382.5	1.087				
C.6	507.8	360.7	1.025				
C.7	530.0	376.4	1.069				
C.8	510.6	362.6	1.030				
C.9	573.9	407.6	1.158				
Median Me, N/mm^2^	Distribution skewness S	Distribution kurtosis K	Confidence interval with 90% probability for population				
			mean strength, N/mm^2^		standard deviation, N/mm^2^		
1.079	0.230	−0.550	1.058–1.113		0.0317–0.0755		

**Table 3:** Compressive strengths and corresponding mean vertical strains.

Compressive Strength, N/mm^2^		
Mean f	Characteristic f_k_	Design f_d_
1.085	0.873	0.565
Mean vertical strains corresponding to		
mean strength ε_f_	characteristic strength ε_fk_	design strength ε_fd_
6.808 × 10^−3^	3.602 × 10^−3^	2.504 × 10^−3^
(9.12%)	(4.43%)	(6.49%)

**Table 4:** Parameters of the deformability model.

	Stress σ, N/mm^2^	Strain ε	Modulus of Deformability D, N/mm^2^	Deformations Characteristic K_i_ = D_i_/f_k_
First branch	0.2f = 0.217	ε_0.2f_ = 1.341 × 10^−3^ (10.4%)	D_1_ = 162	K_1_ = 185
Second branch	0.5f = 0.543	ε_0.5f_ = 2.436 × 10^−3^ (6.47%)	D_2_ = 297	K_2_ = 341
	0	ε_0_ = 6.113 × 10^−4^		

**Table 5:** Parameters of the non-linear model for structure analysis.

	Curve Parameter k_i_, N/mm^2^	Stress σ, N/mm^2^	Strain ε
First branch	$k_{1}=\frac{0.25D_{2}^{2}}{0.2f-D_{2}\varepsilon_{0.2f}}\;$= 121,623	$\sigma_{t1}=\frac{D_{2}\varepsilon_{t1}}{2}=$ 0.182	$\varepsilon_{t1}=\frac{D_{2}}{2k_{1}}\;$= 1.223 × 10^−3^
Second branch	-		
		$\sigma_{t2}=D_{2}\left(\varepsilon_{t2}-\varepsilon_{0.2f}\right)+0.2f=$ 0.327	$\varepsilon_{t2}=\frac{D_{2}}{2k_{2}}+\varepsilon_{f}=$ 1.713 × 10^−3^
Third branch	$k_{2}=\frac{0.25D_{2}^{2}}{D_{2}\left(\varepsilon_{f}-\varepsilon_{0.2f}\right)-0.8f}=$ −29,189		

**Table 6:** Parameters of the linear model for structure analysis.

	Stress σ, N/mm^2^	Strain ε
First branch	0.2f = 0.217	ε_0.2f_ = 1.341 × 10^−3^
Second branch		ε_f1_ = 4.261 × 10^−3^
	f = 1.085	
Third branch		ε_f_ = 6.808 × 10^−3^

**Table 7:** Parameters of tri-linear stress block model for cross-section design.

Parameter	Case ^(1)^		
	1 x ≤ t e ≥ 0.168t = 59.1 mm (Figure 12a)	2 x > t, ε_c2_ < ε_0.2f_ 0.074t = 26.1 mm < e < 0.168t = 59.1 mm (Figure 12b)	3 x > t, ε_c2_ ≥ ε_0.2f_ e ≤ 0.074t = 26.1 mm (Figure 12c)
ε_mu_	3.305 × 10^−3^	-	
ε_c1_	ε_mu_	$\frac{\varepsilon_{m1}}{1-\frac{\delta }{x}}$	
ε_m1_	2.504 × 10^−3^		
ε_0.2f_	1.341 × 10^−3^		
ε_c2_	0	$\varepsilon_{m1}\frac{x-t}{x-\delta }$	
σ_c2_, N/mm^2^	0	D_1_ε_c2_	$D_{1}\varepsilon_{0.2f}+\frac{\left(\varepsilon_{c2}-\varepsilon_{0.2f}\right)\left(f_{d}-\varepsilon_{0.2f}D_{1}\right)}{\varepsilon_{m1}-\varepsilon_{0.2f}}$
χ = ε_m1_/ε_mu_	0.695		
ξ = ε_0.2f_/ε_mu_	0.372	-	
ρ = ε_0.2f_/ε_m1_	-	0.536	
θ = 0.2f/f_d_	0.384		
δ = t(1 − χ), mm	-	107.3	
βx, mm	0.332x	$\frac{5.76\times 10^{-6}x^{3}+2.14\times 10^{-3}x^{2}+25.4x-6.04\times 10^{3}}{37.2\times 10^{-6}x^{2}+0.152x-28.9}$	$\frac{35x-9.83\times 10^{3}}{0.2x-43.8}$
x	$\frac{0.5t-e}{\beta }$	βx + e − 0.5t = 0	
N_Rd_, kN/m	0.339x = xf_d_{1 − 0.5[χ(1 − θ) + ξ]}	$\frac{37.2\times 10^{-3}x^{2}+152x-28.9\times 10^{3}}{x-\delta }$	$\frac{200x-43.8\times 10^{3}}{x-\delta }$

^(1)^ x in mm is a distance between the neutral axis and the most compressed edge of the cross-section.

**Table 8:** Parameters of the model with equivalent rectangular stress block for cross-section design.

Parameter	x ≤ t/κ (Figure 12a)
κ	0.67
η	0.9
α	0.335
X ^(1)^	$\frac{t-2e}{\kappa }$
N_Rd_	ηf_d_(t − 2e)

^(1)^ real extent of compressive stress.

**Table 9:** Parameters for determining the reduction factor for the examined structure.

Parameter	
n_ϵ_(ϵ_u_)	0.601
m_ϵ_(ϵ_u_)	0.397
e_cr_/t	0.160
e_cr_, mm	56.45
Φ_cr_	0.601
α	0.745

## Data Availability

The original contributions presented in the study are included in the article, further inquiries can be directed to the corresponding authors.
